# Sensitivity and Performance of Uncooled Avalanche Photodiode for Thermoluminescent Dosimetry Applications

**DOI:** 10.3390/s24196207

**Published:** 2024-09-25

**Authors:** Piotr Sobotka, Karol Bolek, Zuzanna Pawłowska, Bartłomiej Kliś, Maciej Przychodzki, Krzysztof W. Fornalski, Katarzyna A. Rutkowska

**Affiliations:** 1 Faculty of Physics, Warsaw University of Technology, 00-662 Warsaw, Poland; bartlomiej.klis.stud@pw.edu.pl (B.K.); maciej.przychodzki.dokt@pw.edu.pl (M.P.); krzysztof.fornalski@pw.edu.pl (K.W.F.); katarzyna.rutkowska@pw.edu.pl (K.A.R.); 2 Faculty of Electronics and Information Technology, Institute of Microelectronics and Optoelectronics, Warsaw University of Technology, 00-662 Warsaw, Poland; karol.bolek@pw.edu.pl; 3 Central Laboratory for Radiological Protection (CLOR), 03-194 Warsaw, Poland; z.pawlowska@clor.waw.pl

**Keywords:** low optical signals, photomultiplier, avalanche photodetectors, thermoluminescent dosimeters

## Abstract

Detecting extremely low light signals is the basis of many scientific experiments and measurement techniques. For many years, a high-voltage photomultiplier has been the only practical device used in the visible and infrared spectral range. However, such a solution is subject to several inconveniences, including high production costs, the requirements of a supply voltage of several hundred volts, and a high susceptibility to mechanical damage. This paper presents two detection systems based on avalanche photodiodes, one cooled and the second operating at room temperature, in terms of their potential application in thermoluminescent dosimeter units. The results show that the detection system with an uncooled photodiode may successfully replace the photomultiplier tube commonly used in practice.

## 1. Introduction

Radiation that is either electromagnetic (specifically, X- and gamma-rays) or of a particulate nature (for example, beta and alpha particles) may possess sufficiently high energies to ionize atoms and molecules. This may offer many useful applications in industry and medicine, in the field of radiation protection when detecting the dose of ionizing radiation, for example. This is important because high levels of ionizing radiation can exert a harmful impact on both the environment and human health [1,2,3]. These adverse effects, occasionally revealed immediately after the exposure, may also manifest as long-term consequences for strongly and chronically exposed individuals. For this reason, accurate, robust, and timely information on the dose or dose-rate of ionizing radiation for daily-based operations and decision-making on protective and emergency actions must be provided. This is the “Holy Grail” of modern radiation protection worldwide. 

Dosimetry, allowing for (both natural and artificial) ionizing radiation measuring and monitoring, plays an important role in workers’ and civil protection, including those in the medical, industrial, agricultural, mining, aviation, space, nuclear, and research sectors [4]. Various measurement and signal processing methods may be adopted for this purpose, utilizing different types of detectors (including active and passive ones) with their advantages, drawbacks, and limitations [5,6]. For example, active detectors in the form of ionization chamber-type dosimeters, Geiger-Mueller counters, scintillation detectors, as well as semiconductor, photoelectric, and other integrated sensors [4], are characterized by the lack of versatility of their use in any conditions. This is mainly due to the specific design and additional requirements for their operation (including specific power sources and supplies, controlling systems, insulation covers, electric wiring, etc.). In fact, active measurements typically require electronic circuits to be permanently located in the regions where they are performed, exposing systems to possible damage or interfering with their performance. All this makes active dosimeters bulky and costly, and their operating lifetimes limited to long-term dose/dose-rate measurements. On the other hand, passive detectors do not generally allow for real-time measurements, recording a signal that must be subsequently retrieved and converted to the dose by additional equipment (mostly in laboratory conditions).

The passive (or, in other words, integrating or delayed-reading) devices allow the measurement of a cumulative dose of ionizing radiation through the entire course of a device’s exposure. Passive devices are successfully utilized in occupational and personnel dosimetry, radiotherapy, and research, as well as for environmental monitoring, controlling food irradiation, and, in general, wherever legal dose recording and compliance with regulations are needed. Contrary to active dosimeters, passive dosimeters are characterized by low cost, robust structure, compact size, and constant power-free operation, making them attractive for these applications, where only the integrated dose information is essential and dosimeters are expected to work continuously for an extended period of time. Effectively, thermo- and optically stimulated luminescence [7,8], currently one of the most used phenomena when it comes to passive dosimetry (known as TLD and OSLD, respectively), do not provide immediate measurement results and require some finite time and special equipment for the readout and erasing information of the gathered dose [9], e.g., the precise a priori establishment of a proper calibration curve(s). Such dosimetric measurement techniques, widely used for radiation dose monitoring [10], although subjected to strong criticism [11,12], are accomplished using two main system components–namely, (i) dosimeters (i.e., inorganic crystalline materials recording the total absorbed dose of ionizing radiation and possessing luminescent properties) and (ii) readers with a module for heating or illuminating dosimeters (to release electrons from the trap states) and equipped with detectors measuring the optical signal generated in the luminescence process and proportional to the total absorbed radiation dose accumulated by the dosimeter throughout its exposure. Dosimeters (sometimes referred to as detectors [13] though we have not used the term “detector” in this paper for the sake of clarity) are specifically designed and synthesized to effectively measure different types of ionizing radiation originating from X- to gamma electromagnetic waves, as well as from neutron and beta exposure. The major advantages of luminescence dosimeters are their small sizes, low cost, high sensitivity, simple signal-dose relationships, wide dose linearity range, high precision and accuracy (when specific protocols are used), reusability, and resistance to high humidity and magnetic fields [11]. Specifically, in the well-established and widespread technique of TLD, which of the foremost interest in this paper, the most common thermoluminescent dosimeters (available in various forms including powder, chips, pellets, rods, and ribbons) are made of lithium fluoride (LiF), lithium borate (Li_2_B_4_O_7_), calcium sulfate (CaSO_4_), calcium fluoride (CaF_2_), aluminum oxide (Al_2_O_3_) doped with specific impurities called activators (like Mg, Ti, Cu, Na, P, Dy, Mn, Tm, Tb, B, Si, Cr, and C). Some standard commercial dosimeters are currently available, the most famous [sold, e.g., by Thermo Fischer Scientific (Waltham, MA, USA) and Radcard (Cracow, Poland), respectively] being LiF: Mg, Ti (TLD-100, MTS-N), LiF: Mg, Cu, P (TLD-700H, MCP-N), Al_2_O_3_: C (TLD-500), CaSO_4_: Dy (TLD-900), and Ca: F_2_: Dy (TLD-200). However, each of these dosimeters only applies within specific radiation dose ranges, depending on its linearity, fading, and reproducibility. The dosimetric characteristics of some thermoluminescent dosimeters (TLD), including data regarding the linear dose range (typically from 1 μGy to 10 Gy, as it is for the TLD-700H, and extendable down to 50 nGy and up to 10 kGy for different materials), the wavelength of the maximum luminescence emission (within the UVA or short VIS spectral range and specifically at 370 nm for the TLD-700H), and the fading rate (down to 3% per year for the TLD-700H and TLD-500) may be found in [10,14]. Continuous research is still performed to develop new TL materials, including those that demonstrate high sensitivity and linearity for a wide range of doses, apply in high-energy dosimetry, or perfectly mimic human tissue [15,16,17,18]. Recently, enhanced thermoluminescence (attributed to plasmon-enhanced emission) has been obtained by introducing silver nanoparticles into the LiF matrix [19]. When proposing new alternatives, it is essential to remember that the requirements for dosimeters and their usage, including the TL ones, are internationally standardized as described by the International Electrotechnical Commission (IEC) [20] and the International Organization for Standardization (ISO) [21] documents.

After irradiation, the TLDs are analyzed through the reading system, which consists of a heating module (containing a heating element or coil using heated nitrogen gas or infrared light) and a photodetector [7,10]. The photons emitted during the heating process (correlated to the absorbed dose, or precisely speaking, the total radiation energy absorbed and deposited in the TL material [8] obtained by the appropriate calibration curve, which is usually established before readout) are typically collected using a vacuum photomultiplier tube (PMT). Such a photonic device, still the most frequently used for the detection and measurement of any photon-emitting process in many fields and applications, can measure relatively small amounts of electromagnetic radiation and notably even single photons (in so-called Geiger mode), thanks to the amplification process [22]. Specifically, the PMTs are installed in the TLD readers such as the Harshaw (Thermo Fisher Scientific Inc., Waltham, MA, USA), Risø (DTU National Laboratory, Roskilde, Denmark), Lexsyg Smart/Research (Freiberg Instruments GmbH, Freiberg, Germany), and RE (Mirion Technology/RADOS, Atlanta, GA, USA), to mention some model names currently used and commercially available. The advantages of PMTs are high gain, wide bandwidth, a large photosensitive surface, and relatively low dark current. Moreover, the highest quantum efficiency (QE) of purposely selected PMTs (manufactured by, e.g., Electron Tubes or Hamamatsu [22]) may be achieved within the spectral range of the electromagnetic waves emitted by the TL materials (which is around 400 nm [10,13]). The main disadvantage of photomultipliers is that any spurious signal is also amplified, leading to noise enhancement. Another inconvenience is their susceptibility to mechanical damage and their high voltage requirements (as high as or even above 1 kV). For this reason, they are not suitable for mobile applications, regardless of some portable versions of dosimetric readers (capable of reading out the TLD at the place of exposure) equipped with PMTs being reported (e.g., PorTL^®^, RadPro TLD Cube) [23,24,25]. Furthermore, the development of PMT is relatively slow, as current solutions have reached technological limits [22,26]. The last substantial disadvantage is the price, around a few thousand euros. 

The alternatives to typical single- and multi-anode dynode photomultipliers (PMTs) include microchannel-plate PMTs, hybrid (avalanche) photodetectors [26], and silicon photomultipliers (SiPMs). The latter have been successfully used in time-correlated single photon counting [27], time-of-flight positron emission tomography [28], fluorescence microscopy [29], scintillation readout [30], and calorimetry [31], replacing detecting units based on PMT. SiPM detectors, also known as Multi-Pixel Photon Counters (MPPCs), comprise an array of reverse-biased semiconductor avalanche photodiodes (APDs). APDs are more sensitive than other semiconductor photodetectors and are characterized by high gain, fast response, low dark current, and high sensitivity while operating under a specific bias voltage (above the breakdown one). The state-of-the-art designs of APDs based on group IV materials and a recent overview of non-planar APDs may be found in [32] and [33], respectively. The spectral response range of APD (including integrated and single-photon avalanche diodes, SPADs) is typically within 200 to 1150 nm (with a possibility of extending the spectral range to the short- [34] and mid-infrared [35,36]), depending on the specific diode construction and the semiconductor material they are made of, and hence APDs are becoming popular for general use [37,38,39]. Since each diode can detect only one photon at a time, as already said, the structure is duplicated by connecting all the diodes together, forming the SiPM. In such an arrangement, such a solution enables the simultaneous detection of many single photons, and the output signal could be considered as being proportional to the absorbed amount of ionizing radiation, as it is in TLD readers based on PMTs [25]. In recent years, there has been significant progress in research on improving the parameters of such detectors, including reducing dark current, after-pulsing, and crosstalk, which have been the main weaknesses so far. Specifically, NUV-HD (near-ultraviolet high-density) applications are particularly attractive, achieving perfect parameters in the spectral range characteristic for dosimetry measurements [40]. Another interesting solution for photon detection in the NUV, in the form of Si-based APD with photon-trapping structures, has been proposed [41]. 

Due to the great interest, the commercially available SiPMs have become more cost-effective, and currently the price oscillates around single hundreds of euros, depending on whether the photodetector unit is equipped with a thermoelectric cooler. Importantly, it has been demonstrated that the sensitivity of APDs (including SPADs) has reached a level at which they could easily replace PMTs in some applications. In this regard, our work aims to demonstrate that the development of semiconductor avalanche diodes and SiPM technology allows for their successful application in thermoluminescent ionizing radiation detectors, thereby replacing the photomultipliers used in TLD readers so far. The use of avalanche photodiodes is very promising because their design, size, weight, shock resistance, and price are attractive compared to PMTs. Such features may be remarkably beneficial when used in miniaturized portable devices, which seem to be the prevalent solutions for the future. Even if the judgment of whether usage of APD/SiPM is better than that of PMT strongly depends on the individual application, the rapid development of APD (SPAD) technology in recent years is noticeable. In contrast, the development of PMTs reached a mature level a long time ago. Thus, one of the primary motivations for our studies, and as we believe to be the overall approach for developing the detection systems, is the replacement of PMTs, which are still commonly in use for low-intensity light signals. Specifically, encouraged by our previous achievements demonstrating the possibility of applying a semiconductor photodetector to the effective measurement of a TL dosimetric signal [42], a step forward is made by performing similar measurements using uncooled avalanche photodiodes in an SiPM configuration. The primary motivation is maintaining the same performance as TLD measurement tools but applying much cheaper instrumentation when compared to PMT and cooled SiPM. Even if the main concern is focused on the avalanche diode operating at room temperature, additional measurements have been performed with the use of a cooled SiPM (with its quantum efficiency matched to the luminescence wavelength, which was not the case in our previous work [42]) for comparison. All measurements are confronted with those obtained from TL reading units based on PMTs.

To the best of our knowledge, ours is the first demonstration that the uncooled SiPM system is sensitive enough to be unequivocally used in thermoluminescent dosimeters, allowing for the replacement of PMTs in TLD readers in the near future. The selection of the particular SiPM detectors to be tested was mainly defined by the price and accessibility criteria. In addition to commercial availability, our primary considerations when choosing specific diodes to be used in these studies were the spectral characteristics of the signals to be measured, which are related to the bandwidth and peak of the spectral sensitivity of the diodes. Our solution seems to be much more realistic and practically applicable than the idea of a light-weight battery-operated TLD reader (equipped with a G-1117 Hamamatsu semiconductor photodiode) proposed as a convenient solution for a quick check of TL dating and dosimetry studies and preliminary radiation measurements (before a more precise one is made later in laboratory conditions) [43]. Importantly, further development in our research regarding the application of avalanche diodes allows us to think about their use in routine measurements, not just in TL but also in OSL dosimetry.

## 2. Materials and Methods

This work compares the performance of two semiconductor photodetectors (cooled and uncooled) when applied in a measurement system equivalent to a reader unit used in thermoluminescence dosimetry. The tests were performed using commercial MCP-N ionizing radiation dosimeters manufactured by Radcard (formerly TLD Poland, Cracow, Poland) [44]. The latter, made of lithium fluoride homogeneously doped with magnesium, copper, and phosphorus (LiF: Mg, Cu, P), were in the form of pellets with a diameter of 4.5 mm and a thickness of 0.9 mm. The batch of pellets selected for this study had a sensitivity spread of around 4% (which is below the dosimetric accuracy level postulated in dosimetric protocols [45]). They were irradiated with the known personal dose equivalents (H_p_(10)) equal to 1 mSv, 5 mSv, and 10 mSv, respectively. These test values were chosen considering the maximum effective dose limit of 20 mSv per year for people working in radiation conditions, and the limit of 1 mSv for the general population, as stated by the requirements for people and the environment [20], as well as in occupational [46] radiation protection, standardized by the International Atomic Energy Agency (IAEA).

The TL dosimeters were exposed to ionizing radiation in the Central Laboratory of Radiological Protection (CLOR), Poland, an accredited laboratory for the calibration of dosemeters. Before exposure, the TL dosemeters were annealed in a nitrogen atmosphere at 270 degrees Celsius for 30 s. Next, the irradiation was done using a certified ^137^Cs source in a well-defined radiation field, as specified by the norm ISO 4037-1:2019 [47]. The dosimeters were placed on a solid PMMA slab phantom 2 m from the radioactive source to the middle of the pellet and exposed for a set duration. The exposition time was adjusted to the dose-rate at the position of the pellets inside the holder to result in precise H_p_(10)s of 1 mSv, 5 mSv, and 10 mSv, respectively. The uncertainty of the H_p_(10) was estimated based on a regular beam dose-rate characterization via ionization chambers, with a correction for caesium decay. The time for the irradiator shutter to release and stop the gamma-ray beam was negligible compared to the exposure time. Additionally, the reading procedure was performed no longer than one day after the exposure. Thus, the dose from background radiation was negligible. Finally, the uncertainty of the H_p_(10) to which the TL dosimeters were exposed was equal to 1.02%. Thanks to that procedure, one can establish a calibration curve, namely the simple function between doses and counts, which will be useful for future dose measurements. Please note that precise calculations of the calibration curve’s parameters are crucial for correct dose assessment. 

The reading procedure of TL dosimeters consists of two phases, i.e., initial preheating, which takes 5 s, and proper heating at 250 degrees Celsius for 15 s. During heating, the TLD emits light with a maximum intensity at the wavelength of 360 nm [13,48] and with its total radiant power proportional to the absorbed dose of the ionizing radiation. The measurements of emitted light were made using the two SiPM detectors under investigation: (i) an S13362-1350DG from Hamamatsu [49], cooled to −20 degrees Celsius, (ii) a MICROFC-SMTPA-30035-GEVB from Onsemi [50], operating at room temperature. The measurements were then compared to the reference data obtained from the photomultiplier tube (taken from the commercial TLD RADOS reader). A list of the parameters of both systems (based on cooled and uncooled avalanche photodiodes) is presented in Table 1.

The original system, adopted for reading luminescence signals with the use of the SiPM detectors, was based on a transimpedance amplifier, an adjustable discriminator (to eliminate the noise generated by the detector), and a fast analog-to-digital converter (ADC). Such a configuration appeared to be the best and the simplest among the many tested solutions to be applied as a TL reader based on SiPMs. In the case of the Hamamatsu detector, the device (module) was equipped with its own temperature control circuit, a high-voltage power supply circuit (with bias voltage stabilization), and a signal preamplifier (see Ref. [49] for more details). While the module operates just by connecting it to an external power supply of ±5 V, the connector to the transimpedance amplifier and ADC are the only ones to be prepared. In the case of the Onsemi detector (with its datasheet given in Ref. [50]), the custom front-end electronic circuit was built to read out the signals. Such a circuit consists of a proper power supply that provides a stabilized voltage bias slightly above the breakdown on the detector diode and signal processing unit. The exact designs of the circuits mentioned above are the subject of the Polish patent application (application of invention number P. 443362 [51]), and, thus, more details on the exact schemes or elements used cannot be given in this paper.

At this point, it is essential to remember that the photodiodes under test were operating in the Geiger mode, GM (i.e., identified with single-photon detection), with the bias voltage of the diode exceeding the threshold voltage of the avalanche breakdown (and the increased high gain controlled by the quenching circuit), in which the internal noise turns out to be vanishingly low. The GM implies that multiple photons counted by the detector at precisely the same time cannot be distinguished. This issue becomes relevant when the proposed TL reader is applied in cases of high doses, for which a nonlinear response of the detector at the high luminescence signal may be expected. It is crucial to underline that in the tests performed in this study, only low-dose signals were measured, with the GM applicable for all of the light intensities registered. It is important because, thanks to the linear mode appropriate to the system under consideration, it is easy to calibrate the detector response as a function of the ionizing radiation dose. Of course, presumably, the linear mode of the system could also be applied to cover the high-dose ranges. In the specific case of our signal processing setup based on a transimpedance amplifier, it is possible to switch to the linear mode by lowering the APD bias voltage, but it is rather undesirable (and not necessary in the studies described in this paper).

To measure the signal-to-noise ratio (which is the most appropriate parameter to define the dynamics of the measuring system and the possibility of using it in practice), the test setup was built to reproduce the measurement conditions of the TLD reader as accurately as possible [42]. The setup consisted of an LED emitting light pulses with a frequency of 10 Hz and a wavelength of 420 nm. The LED was placed in a special housing that allowed us to change the light’s intensity using different apertures. With this, it was possible to specify the signal-to-noise ratio (SNR), defined here as the average value of the signal when the testing light was on to the mean signal value when the testing light was off. Moreover, to show how large the fluctuation of the measurement signal is in relation to the measurement value itself, the variation coefficient (VC), defined here as the standard deviation divided by the value of measurement, has been calculated. For both detection systems (i.e., with the cooled and uncooled diodes), the VC was about 3% and the SNR ranged from about 2 to about 20 (depending on the photodiode and increasing for higher doses). The latter value is not always particularly high when compared to the SNR value obtained for the typical PTM, being in extreme cases as much as one order of magnitude higher (and with the SNR ≥ 10 considered as “excellent” with a general rule of thumb [52]). However, it should be remembered that the aim of this study was not to directly compare the performance of SiPM and PMT detectors, but to demonstrate that the SPADs are good enough for TL reading within the considered dose range (≥1 mSv and ≤10 mSv), with some benefits resulting from their characteristics (such as low price, small dimensions, integration possibilities, etc.).

It is worth noting that when measuring the luminescence signal, the background noise was noticeable, but it was not subtracted from the measured signal. Information about the background noise may be retrieved based on the linear regression performed to fit the experimental data (i.e., from the values of the intercepts).

When considering the speed characteristics and performance of the avalanche photodiodes, it is worth mentioning that there are a lot of works on time-of-flight detectors and other solutions for which speed characteristics are relevant. Such a feature is not critical in the considered case because the measurements are relatively long (with a duration of about 15 s), the light intensity is low, and most importantly, the integrated signal is taken into account and used for the further dosimetric calculations investigated in this paper. For this reason, the speed characteristics of the SiPM detectors chosen for the tests were not considered.

## 3. Results

**Figure 1:**
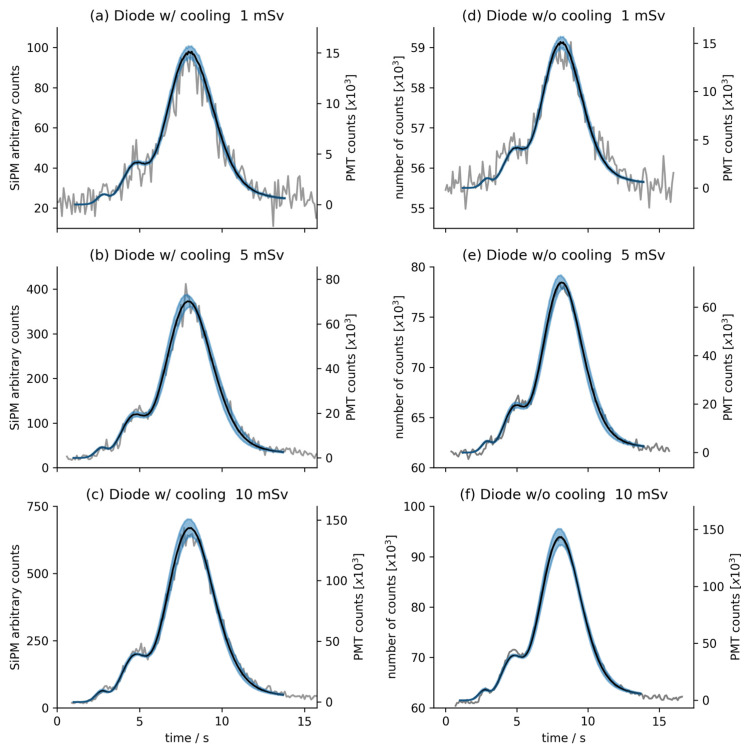
The signal read from the TLDs irradiated with the dose equivalents of 1 mSv, 5 mSv, and 10 mSv. Panels (**a**–**c**) show the results obtained for the cooled S13362-1350DG SiPM detector, while panels (**d**–**f**) represent the uncooled MICROFC-SMTPA-30035-GEVB SiPM detector results. The vertical axes on the left side correspond to the data collected by the investigated photodiodes (gray lines), and the axes on the right side refer to the reference measurements from the PMT. Specifically, the black lines show the average value of eight photomultiplier measurements for a given dose equivalent. The area of uncertainty with a half-height equal to the one standard deviation of these measurements is marked in blue.

Figure 1 shows the results of the thermoluminescence measurements of MCP-N dosimeters made in the same measurement system when using three different photodetectors. Specifically, the gray curves in graphs from Figure 1a–c present results for three dose equivalents (of 1 mSv, 5 mSv, and 10 mSv, respectively) obtained using the cooled Hamamatsu S13362-1350DG SiPM detector (also named as diode w/cooling), and the gray curves in graphs from Figure 1d–f show the same dose equivalent measurements obtained with the Onsemi MI-CROFC-SMTPA-30035-GEVB uncooled SiPM detector (also named as diode w/o cooling). Additionally, all graphs include average data from the eight MCP-N TLD photomultiplier tube measurement series (black lines) with uncertainty within one standard deviation (marked in the blue area). Since the sensitivity of the PMT is much higher than that of the tested photodiodes, it can be assumed that the obtained spread describes the accuracy of the irradiated dose. For the dose equivalents of 5 and 10 mSv, the measurement spread obtained from both SiPMs is within the standard uncertainty limit.

**Figure 2:**
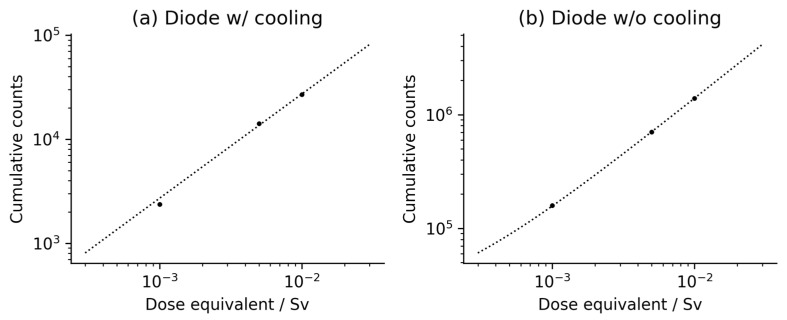
Data of dose equivalent measurements for (**a**) Hamamatsu S13362-1350DG detector (Diode w/cooling), and (**b**) Onsemi MI-CROFC-SMTPA-30035-GEVB (Diode w/o cooling) for low doses up to 10 mSv. Parameters of the linear function fitting (dotted lines) are given in Table 2. Please note that uncertainties of both the cumulative counts and the dose equivalents are too small to be noticed on the graphs.

In addition to the results of the measurements presented, the relation between the nominal dose and the cumulative counts obtained from both (cooled and uncooled) SiPMs was determined. The results are presented in Figure 2 and Table 2. To avoid any bias in future dose assessments, three methods of the linear best fit were used: (i) the classical least square method (LSM), (ii) the total variance method (TVM) [53], and (iii) the robust Bayesian regression method [54] for independent validation. In all cases, the linear function fits the experimental data points well, which is important for the calibration curves necessary for further experimental investigations. Please note that the curvature of the fitted line in Figure 2b is only an artifact related to the double logarithmic scale used in the graph and the relatively high intercept value in this case. It may be stated that both of the investigated photodetectors are characterized by high linearity (with the determination coefficient R^2^ > 0.997, depending on the case and the method), which is essential for determining the calibration factor of the photodetector when applied in a feasible dosimetric reader. All of the fitting parameters are presented in Table 2.

## 4. Conclusions

The experimental results presented in this paper indicate that the sensitivity and performance of uncooled avalanche photodiodes can be considered sufficient for the routine measurements of ionizing radiation with the use of thermoluminescence dosimeters, with no evidence of nonlinearity within the 1–10 mSv dose equivalent range under consideration. This finding is important because all three statistical methods (Table 2) show very consistent results and a lack of outliers, which could easily establish a calibration curve for further practical dosimetric measurements. At the same time, the obtained results confirm the great potential (which is still underutilized when it comes to applications in passive dosimetry) offered by the avalanche photodiodes equipped with active cooling systems. The measurements described in this paper clearly show that commercially available avalanche photodiodes (here in an SiPM configuration) allow for results comparable to those obtained with the use of photomultipliers. This is a huge step forward in low-level light and single-photon detection (here regarding dosimetric measurements but with potential photonic applications extending well beyond that), as using semiconductor elements will significantly reduce production costs. Specifically, avalanche photodetectors are revealed as essential components of integrated photonics, having the potential to revolutionize the measurements of low light signals, as they are fast, highly sensitive, and compatible with silicon photonics platforms. Such photodetecting elements themselves are several times smaller than PMTs, at least two orders of magnitude cheaper, and, like most semiconductor elements, shock resistant. They are still experiencing technological development, which is contrary to the tube photomultipliers, the physical basis of operation and the production technology of which have not changed since the beginning of their production several dozen years ago. Based on the authors’ experience, it is certain that APDs, SPADs, and SiPMs will be gaining even more attention in the future, so it is reasonable to explore more and more areas for their application. 

In future studies, the authors plan to continue their work, focusing on using cheap uncooled avalanche photodiodes for signal detection from TLD dosimeters. Moreover, they intend to incorporate the use of semiconductor photodetectors to measure ionizing radiation when facilitating optically stimulated luminescence (OSL), a methodology in increasing use in the field of passive dosimetry. In fact, OSLD, with its many similarities to TLD (such as the physical forms of the dosimeters and their basic measurement mechanisms), has recently emerged as a technique with vast potential, with its all-optical technological advantage over the well-established TLD and not requiring heating of the sample and thus simplifying the required equipment. Due to the optical nature of stimulation, OSLD may widen the areas of applicability, taking into account that recent commercial readers are constructed to perform measurements of both TL and OSL signals. 

## Figures and Tables

**Table 1:** The most critical parameters of the investigated SiPM detectors, with values taken from the datasheets provided by the manufacturers [49,50].

Name	Cooling System	Active Area /mm^2^	Microcell Size /µm	Spectral Sensitivity Bandwidth (Peak) /nm	Photon Detection Efficiency (PDE) at Peak/%	Dark Count /kcps
Hamamatsu S13362-1350DG	Yes	1.3 × 1.3	50	320–900 (450)	40	13
Onsemi MICROFC-SMTPA-30035-GEVB	No	3 × 3	35	300–950 (420)	41	300

**Table 2:** Parameter values obtained after the linear function fitting to the datapoints from Figure 2 to establish final calibration curves. Three statistical methods were used: the classical Least Square Method (LSM), the Total Variance Method (TVM) and the Robust Bayesian Analysis (RBA) [43]. All uncertainties represent one standard deviation.

Type of Diode	Method of Fitting	The Slope/Sv^−1^	Intercept	R^2^
w/cooling (Figure 2a)	LSM	(2719 ± 120) × 10^3^	−7 ± 776	0.99806
	TVM	(2811 ± 118) × 10^3^	−415 ± 282	0.99823
	RBA	(2752 ± 259) × 10^3^	377 ± 984	0.99754
w/o cooling (Figure 2b)	LSM	(1374 ± 9) × 10^5^	19,524 ± 5752	0.99996
	TVM	(1366 ± 9) × 10^5^	22,799 ± 1516	0.99996
	RBA	(1366 ± 161) × 10^5^	22,799 ± 2875	0.99992

## Data Availability

The data presented in this study are available on request from the authors.

## Abbreviations

The following abbreviations are used in the manuscript:

ADC	Analog-to-Digital Converter
APD	Avalanche Photodiodes
CLOR	Central Laboratory for Radiological Protection, Warsaw, Poland
GM	Geiger mode
IAEA	International Atomic Energy Agency
IEC	International Electrotechnical Commission
ISO	International Organization for Standardization
LSM	Least Square Method
MPPC	Multi-Pixel Photon Counter
NUV-HD	Near-Ultraviolet High-Density
OSL	Optically Stimulated Luminescence
OSLD	Optically Stimulated Luminescence Dosimetry/Dosemeter
PMT	Photomultiplier Tube
QE	Quantum Efficiency
RBA	Robust Bayesian Analysis
SiPM	Silicon Photomultipliers
SNR	Signal-to-Noise Ratio
SPAD	Single-Photon Avalanche Diodes
TL	Thermoluminescence/Thermoluminescent
TLD	Thermoluminescent Dosimetry/Dosemeter
TVM	Total Variance Method
w/	with
w/o	without
VC	Variation Coefficient
VIS	Visible (light)
